# Non-Thermal Processing Technologies in Food Industries

**DOI:** 10.3390/foods15101677

**Published:** 2026-05-11

**Authors:** Xinyu Yuan, Yihan Fang, Yuhan Diao, Beibei Wu, Xiang Wen, Anindya Nag, Yan Liang

**Affiliations:** 1 School of Food and Pharmacy, Zhejiang Ocean University, Zhoushan 316022, China19818036195@163.com (Y.D.); 19858064298@163.com (B.W.);; 2 Faculty of Electrical and Computer Engineering, Technische Universität Dresden, 01062 Dresden, Germany; anindya.nag@tu-dresden.de

**Keywords:** non-thermal, food, food processing, high-pressure processing, pulse electric field, ultrasound technology

## Abstract

The paper presents a substantial review of the use of non-thermal technologies in the food industry. In contrast to thermal processing, non-thermal techniques have been very effective in improving and maintaining the nutritional composition of food items. As an alternative to thermal processing, various ambient conditions, including energy consumption, exposure time, and other parameters, have been considered for non-thermal processing to achieve reduced microbial load in food products. The paper provides a comprehensive overview of fundamental non-thermal processing techniques, including high-pressure processing, pulsed electric field, ultrasound, and cold plasma, used to maintain food quality and extend shelf life. The paper also emphasizes certain factors, such as novel hybrid techniques, which can enhance key parameters, including customer acceptance, sustainability, interdisciplinary collaboration, and industrial recommendations. It includes work on product quality, energy consumption, efficiency, and support for the adoption of advanced food processing technologies.

## 1. Introduction

With an ever-increasing global population, a corresponding increase in food production is a constant requirement. The demand for improved food quality has increased significantly following the COVID-19 pandemic [1,2]. This has prompted researchers to employ scientific methods to develop nutritious foods with optimized calorific values. Both the public and media houses have compelled the food industry to develop food with optimal nutrient composition to minimize food-related health issues in humans [3,4]. After the food is marketed, it is also important that it reaches the consumer and maintains its post-prandial quality. Although the quality of the food received does not depend solely on post-prandial actions, it is also influenced by factors such as the breakdown of food constituents, food rheology, and food structure [5,6]. This subsequently affects the individual’s digestive and subsequent metabolic processes [7,8]. Each food structure can be defined as having a particular three-dimensional arrangement of cells or molecules [9,10]. The variation in each arrangement can simultaneously differ in certain properties, such as rheology, texture, flavor, and process stability, at both the macro and microscopic levels [11,12]. To maintain satiety, flavor, food structure, and process stability, researchers have been exploring various processing technologies to formulate food with optimal nutrients and functional attributes [13,14]. These processing technologies can alter the physicochemical and nutritional properties of food, thereby affecting its overall quality and value. Therefore, it is crucial to investigate the food processing technologies employed in the industry.

**Figure 1:**
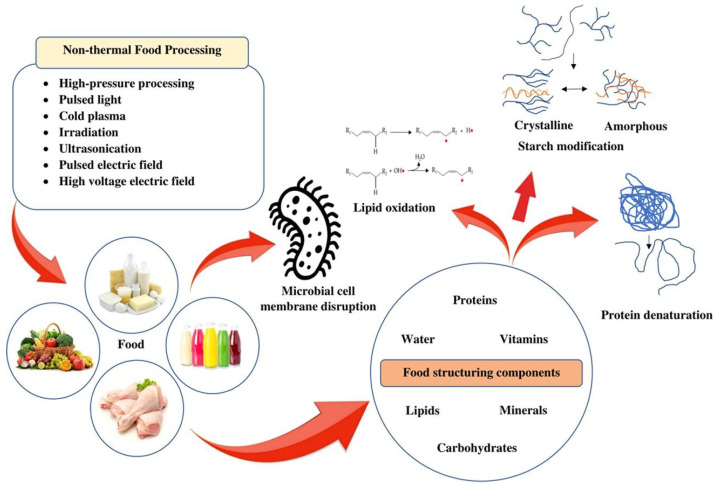
Representation of the use of non-thermal food processing technologies for structural changes in food components [24].

Food processing has been carried out using a variety of traditional and non-traditional methods. Among traditional methods, heating is the most common. It alters the food structure by modifying its stability, primarily to prevent food spoilage and maintain microbial stability. The treatment of food with heat induces various physicochemical changes, and severe conditions can significantly affect organoleptic properties. This would be detrimental to heat-sensitive food vitamins, which would subsequently remove bioactive compounds and generate potentially unfavorable compounds, such as advanced glycation end products, keto-aldehydes, glyoxal, heterocyclic amines, and acrylamides [15]. Thus, it is essential to develop non-heat-based methods, such as pulsed electric field (PEF) [16,17], high-pressure processing (HPP) [18,19], cold plasma [20,21], and high-voltage electric field [22,23]. The development of advanced processing technologies has been crucial to producing high-quality food products with minimal change. Non-thermal processing technologies have certain characteristics that offer shorter treatment times and lower temperatures [15]. In recent times, certain non-thermal processing technologies, such as high hydrostatic pressure, have been replacing traditional processes and are poised for commercialization. Structural alteration of food can also be achieved using non-thermal technologies. There is interest in various methods within non-thermal technologies to improve certain attributes, such as digestibility, water-binding capacity, and the modulation of gelation processes. It is essential to analyze the constituents of food components, as these will ultimately influence their technical properties. Additionally, the differences in primary operation and equipment design mechanisms will impact their performance under various operating conditions. Figure 1 [24] shows the schematic diagram of the different non-thermal food processing technologies used to modify the constituents of the food products. It is observed that numerous non-thermal food processing techniques have altered the basic constituents, such as proteins, lipids, and carbohydrates, leading to changes including membrane disruption, denaturation, oxidation, and modification.

Table 1 provides the characteristics of thermal and non-thermal food processing techniques. It is observed that although both techniques offer individual advantages in terms of applications and shelf life, the use of non-thermal processes has been more advantageous in terms of the safety and quality of processed food. With the currently available traditional thermal processing techniques, such as pasteurization, refrigeration, and freezing, some limitations include high energy consumption, loss of heat-sensitive nutritional components, textural changes, and alterations in rheological characteristics.

Additionally, certain techniques, such as thermal processing, result in a loss of hydration, changes in fatty acid composition, and lipid oxidation [25]. The barbequing of certain meats leads to the loss of juices, a reduction in saturated fatty acids, and an increase in polyunsaturated fatty acids. There is an increasing demand for nutritional food with no microbial load and excellent mouthfeel. The use of alternative approaches, such as emerging non-thermal technologies [25,26], has enabled the production of higher-quality food with a reduced microbial load. Optimizing parameters such as food type, processing time, intensity, and conditions has enabled us to achieve the desired physical, chemical, and biological characteristics. The recovery of bioactive substances has also enabled the enhancement of health-linked compounds in minimally processed fruits and vegetables. Certain processes, such as UV light, can also induce the synthesis of anthocyanins, vitamins, carotenoids, polyphenols, and flavonoids under specific conditions, thereby increasing the overall quality, safety, shelf life, and digestibility of food materials [27,28].

**Figure 2:**
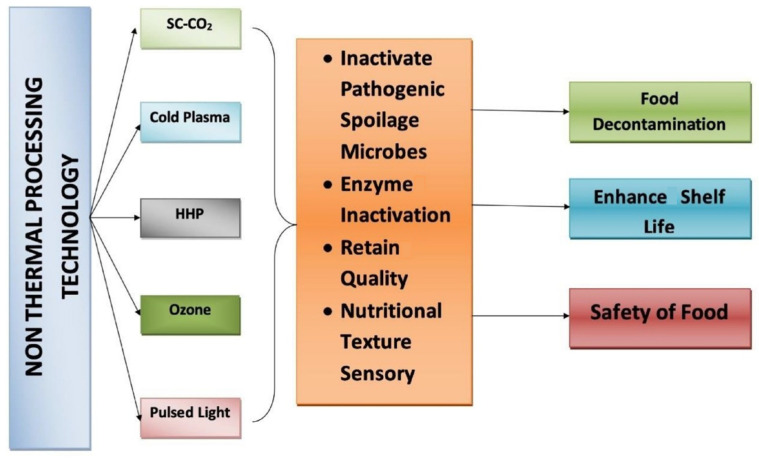
Representation of the performance of the non-thermal food processing technologies to reduce contamination and ensure food safety [25].

The use of non-thermal processing technologies is also beneficial chemically, as they typically affect non-covalent bonds in food molecules, including hydrophobic, hydrogen, and ionic bonds. The processing techniques denature, inhibit, and gelatinize proteins, enzymes, and starch, and destroy microorganisms and pathogenic bacteria [29]. Figure 2 [25] presents the capabilities of various non-thermal food processing technologies to enhance food safety and reduce contaminants.

## 2. Non-Thermal Processing Technologies

With a growing need to maintain food quality during processing for preservation, the use of conventional processing technologies has led to undesirable changes in food. Some irreversible changes, such as the loss of temperature-sensitive nutrients and alterations in food texture, result in the production of low-grade food [27,30]. This necessitates the use of non-thermal processing techniques as a gentler alternative to traditional heat treatments, aiming to minimize the formation of carcinogenic chemical toxicants in the human body [31,32]. The thermal treatment of food also has other effects, such as water loss, lipid oxidation, changes in fatty acid composition, and increased formation of polyunsaturated fatty acids. The increasing demand from customers for fresher food materials with longer shelf life and improved sensory properties has driven extensive research into non-thermal food treatment [33]. Other attributes, such as low energy consumption and the generation of food that can be partially replaced by consumer-friendly, environmentally friendly, and cost-effective technologies, enabled the researchers to develop non-thermal processes to reduce microbial load and maintain sensory and textural characteristics [34,35].

### 2.1. High-Pressure Processing (HPP)

**Figure 3:**
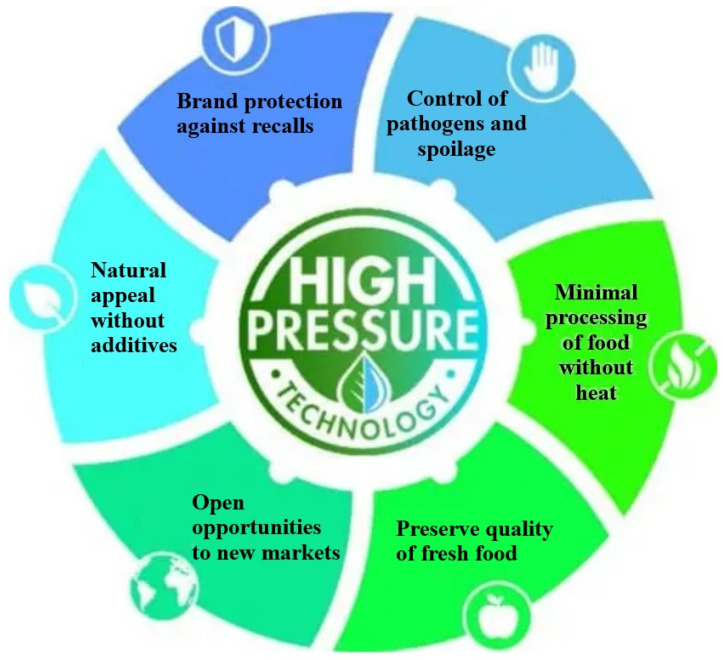
Representation of the advantages of the HPP process to maintain the quality of food products [37].

HPP has been effectively used to eliminate harmful microorganisms and purify food components. The HPP considered in industries uses a common medium, water, to treat the product. The equipment required for HPP has several advantages, including an extended shelf life, superior food safety and quality, and increased yields and efficiency [36]. Figure 3 [37] represents some of the advantages of the HPP technique. Here, the food components are introduced inside a chamber and subsequently pressured with water. It can deal with various types of pathogens, including Gram-negative and Gram-positive bacteria, yeast, and other molds. It also reduces the presence of other pathogens, such as *Salmonella* and *E. coli*. This process has been considered for preserving food for a longer duration. It has been used in various applications, including controlling Listeria monocytogenes in ready-to-eat foods and as an alternative to thermal pasteurization of raw milk. Although this process uses high pressure for a fixed time, the pressure has a minimal effect on taste, texture, and appearance. Normally, the food is subjected to a pressure range of around 200–700 MPa. To deal with certain microorganisms, such as Gram-positive bacteria, a pressure of more than 400 MPa is required [18]. This causes the bacterial cell membrane to break, thereby altering the permeability of the microbial cell wall and membranes. The quality of the nutritional components and texture, as determined by this method, is excellent due to the brief exposure time. The amount of reduction is dependent on the duration and intensity of the applied pressure. For example, it can extend the shelf life of safe, minimally processed products. This technique has been employed at various stages of the food chain, including pre-packaging. It has been deployed on both raw materials, such as milk and fruit juices, as well as on cooked products, including meat and ready-to-eat meals. The amount of contamination of food from the ambient environment is reduced using the HPP technique. This process has been highly efficient for inactivating microbes in various types of food products. The most processed foods include fruits, meat, and dairy products. This process has also been used for microbial inactivation, as shown by Bulut et al. [38]. They have used HPP against *E. coli* in liquid food samples, followed by storing orange juice at a temperature of −80 °C. HPP was applied at 250 MPa for 900 s. The application of HPP to orange juice reduced the microbial load, accompanied by subsequent changes in pH. Another example is reported by Cap et al. [39], who reduced *Salmonella* levels in meat using high-pressure processing (HPP). The experimental process involved inactivating the *Salmonella* bacteria in the chicken breast using a pressure of 500 MPa for 60 s [40]. Similar studies have been carried out using various food items, including tomato waste [41], grape pomace [42] and egg yolk [43]. In addition to bacteria, HPP has also been capable of extracting and enhancing antioxidants, phenols, and bioactive and functional components from various sources. Although HPP has been widely used, it has several limitations. One major drawback is the high cost of front-end operational equipment, making it less accessible to small-scale processors. It is typically a batch process, which limits throughput and reduces the overall efficiency for large-scale continuous production. Another limitation is the dependence of this method on flexible, water-resistant packaging materials that can withstand extreme pressures, thereby limiting packaging options. The HPP method is also less effective against bacterial spores, so it is necessary to combine it with other hurdles, such as mild heat. This reduces the standalone efficacy of the process. Another drawback is the inability to fully inactivate enzyme activity, which can lead to quality changes during storage. Textural modifications can occur in some foods, particularly in protein-rich solids.

### 2.2. Pulsed Electric Field (PEF)

**Figure 4:**
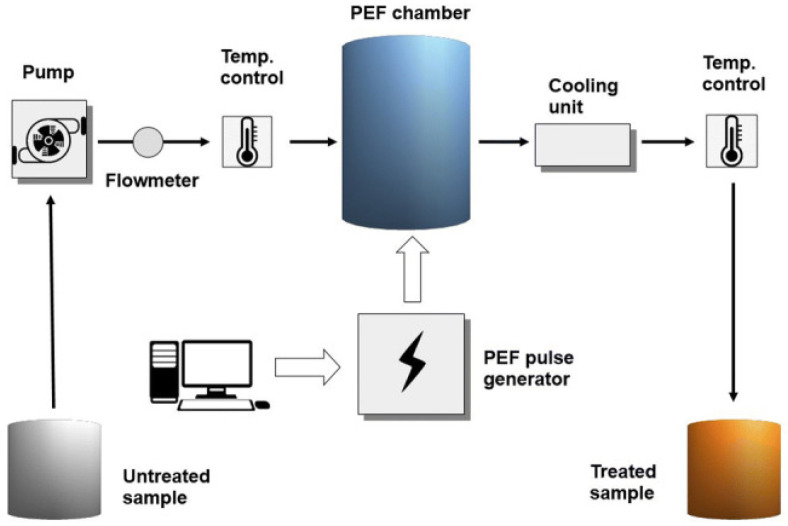
Schematic diagram of the experimental chamber for the PEF process [44].

PEF is an emerging non-thermal technology that can process food materials with high efficiency. This technique involves applying a high-intensity pulse to food items for a very short time. It has been shown to enhance food quality and provide high nutritional value [19]. The treatment of food items involves exposing them to an electric field of 25–85 kV/cm. The exposure is brief, lasting between nanoseconds and a few milliseconds. A typical PEF unit used for food processing is shown in Figure 4 [44]. The PEF process unit consists of a food treatment chamber, a fluid-handling system, a control system, and a high-voltage pulse generation unit. The first component shown in the image can supply high-voltage pulses with the desired shape, duration and intensity. The treatment chambers are divided into separate batches and continuously filled according to the treated food product. The entire process is controlled by a central computer, which assists in setting the parameters, operating the pump and obtaining the data from the probes placed inside the chamber. Some advantages of PEF processing include reduced energy and water use, as well as improved cutting quality and yield during extraction and pressing. Since the probability of heating (other than Joule heating) the food is almost nil due to the short exposure, any permanent chemical changes in the food resulting from the temperature change are eliminated. Although this process has been employed for some time, it has gained widespread use in recent years for microbial inactivation in food products. The treated food is placed between two electrodes, generally made of stainless steel, in the treatment chamber [45,46]. Compared with HPP, this process can treat liquid or semi-solid foods that flow easily [47]. Following the PEF treatment, the cell membranes of the microbes are damaged. In addition to the high intensity of the pulse, the presence of hydrogen peroxide leads to oxidative changes in the bacterial cells’ lipids and proteins. This results in the inactivation of metabolic enzymes, ultimately leading to cell death [48].

The efficiency of this technology primarily depends on the intensity of the applied field, the total exposure time, the temperature, and the energy. The PEF process has been widely used to extend the shelf life of food products. Certain studies, such as those by Preetha et al. [49], have shown reductions in *E. coli* in flowable foods, including pineapple, orange juice, and coconut water. A PEF at 5.6 kV/cm reduced the *E. coli* load to 4.5, 4, and 5.3 log CFU/mL in pineapple, orange juice, and coconut water, respectively. While the microbial load decreases with PEF, larger microbial cells are more easily exposed and affected. Even though smaller microbial cells remain unaffected by this process, it has been effective in deactivating the food spoilage enzymes. In a recent study by Käferböck et al. [50], it was observed that the extraction functional components from microalgae was carried out using PEF with a frequency of 300 Hz and pulse widths of 4–32 µs. The work done by Liu et al. [51] is another significant study that utilizes PEF for unit operations such as dehydration and freezing. They used PEF to reduce carrot drying time. A PEF was applied at 0.6 kV/cm and 0.1 s. The reduction in drying time was achieved by 55% at 25 °C and 33% at 90 °C.

Like the HPP process, PEF also has significant limitations. Firstly, it is primarily effective for liquid or pumpable foods, thus making it unsuitable for solid or highly heterogeneous products. The PEF requires food with sufficient electrical conductivity, which limits its formulation flexibility. This process also requires a high initial investment and significant energy, limiting its adoption in developing food processing industries. Another major technical challenge is to achieve a uniform electric field distribution, especially in large-scale systems. This can lead to inconsistent microbial inactivation. Electrode corrosion and fouling are additional operational issues that increase maintenance costs and shorten equipment lifespan. Similar to HPP, this process is also less effective against bacterial spores, often requiring a combination with other treatments. Post-processing contamination risks persist if aseptic conditions are not maintained. Finally, the scale-up from the laboratory to the industrial level remains complex due to challenges with flow and field control.

### 2.3. Ultrasound Technology

**Figure 5:**
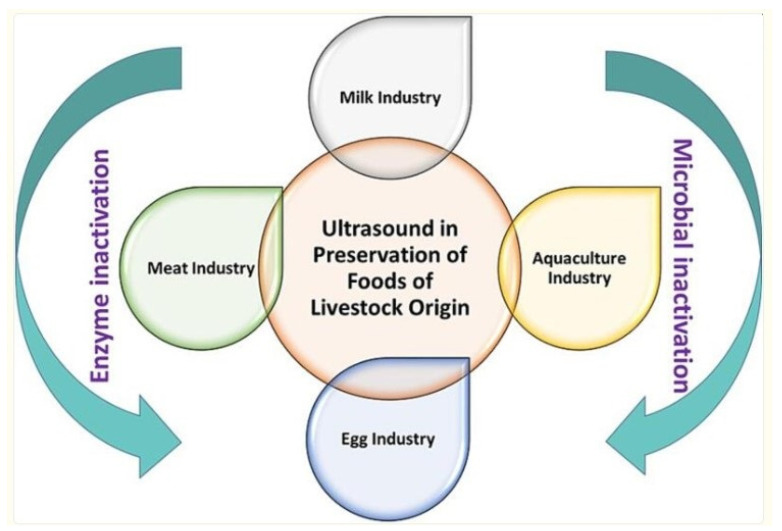
Representation of the impact of ultrasonication on the preservation of foods of animal origin [53].

Although researchers have started using ultrasound technology in the food sector in recent years, it has been a prominent technology in other processing sectors. Ultrasound technology utilizes sound waves with frequencies higher than those of normal human hearing, i.e., 20 kHz [52]. In general, traveling ultrasonic waves generate expansion and compression effects in the medium. The presence of air in the medium leads to the formation of small cavities. The shape and size of these cavities may vary, thus generating a large amount of localized energy. This leads to the formation of local hot spots, subsequently increasing the overall heat and mass transfer rates [38]. This process involves an ultrasonic horn dipped in a liquid food solution and operated at a specific input frequency. It is typically carried out using an ultrasonic bath, where the food material is placed, and sound waves are generated within the bath. The presence of ultrasonic effects achieves the required changes. Figure 5 [53] shows the impact of ultrasonication on the preservation of food of animal origin. Some of the advantages of using ultrasonication for food processing include enhanced effective mixing and micro-mixing, faster energy and mass transfer, reduced thermal and concentration gradients, lower temperatures, selective extraction, smaller equipment size, and faster response to process extraction control [54]. This technique is typically used in organic chemistry to enhance reaction output by increasing heat transfer and mass transfer. Variation in the ultrasonication frequency range alters the shear forces in the medium. While the frequency is inversely proportional to the shear force, the medium-frequency range (20–100 kHz) can produce radical species. This range is considered optimal for various sonochemical-assisted food processes. Since this process sometimes leads to the formation of undesirable chemical radicals and induces undesirable changes, it is important to determine the frequency range for certain food items such as lipids and proteins [55]. Food processing technology that utilizes an ultrasonic process employs a frequency range of 20–100 kHz to extract bioactive compounds, enhance cooking, and intensify synthesis, among other applications. Jadav et al. [56] used ultrasonication to synthesize designer lipids. This method was found to be an excellent alternative to the conventional method for intensifying the yield. The output was around 92% after six hours of reaction. An increased yield was obtained due to the subsequent increase in energy, which resulted in the formation of high-energy spots. This allowed for an increase in the rate of mass transfer, enabling the quick completion of the reaction. It also facilitates the interfacial transfer of molecules, thereby enhancing the overall efficiency of extracting bioactive compounds from plant and animal sources.

Sun et al. [57] demonstrated the use of ultrasonication to extract proteins with superior characteristics. These protein molecules had better particle size, physical structure and power. Ultrasonication at 20 kHz for 30 min facilitates the production of small particles with a larger α-helix structure. Another interesting work can be found in the research by Cheila et al. [58], which employed ultrasonication to adapt a greener approach to extract bioactive compounds from velame leaves. The use of indirect ultrasound waves increased extraction to 94% in 39.5 min. This process has also been proven to be an effective technique for extracting oil from various food items, such as olive fruit, soybeans, and flaxseed [46,47]. This process has also been very effective for the dairy and beverage industries. For example, membrane filtration in cheese-making is performed using ultrasonication [52]. It is used to complete the separation of milk protein from other milk solids. In one of the research projects by Mothibe et al. [56], ultrasonication was used as the initial processing step before apple dehydration. When the drying time was reduced, the dried product achieved a good texture with less water activity.

Although ultrasound technology has served a great purpose as a non-thermal food processing technique, it is also limited by several practical and technical constraints. One major limitation is its poor penetration depth, which makes it unsuitable for bulk processing. Its effectiveness often decreases in large or dense food matrices. The cavitation effects associated with ultrasound technology can also cause undesirable physical and chemical changes, such as nutrient degradation, lipid oxidation, or texture damage. Similar to PEF, scaling up ultrasound systems for industrial use is challenging due to uneven energy distribution and difficulty maintaining consistent cavitation intensity. Equipment erosion due to prolonged cavitation is another operational concern in this process. Another issue is this technique’s inability to achieve sufficient microbial lethality, requiring a combination with other food processing treatments.

### 2.4. Cold Plasma Technology

Plasma is known to be the fourth state of matter after solid, liquid and gas. As kinetic energy increases, solids first change to a liquid, then to a gas. The increase in energy leads to the disintegration of the intermolecular structure, thereby transforming the phase. The increase in the energy for the gaseous phase leads to the ionization of the gaseous molecules [59]. The ionization of gaseous molecules is known to occur in plasma. Plasma treatment is divided into two types: thermal and cold plasma. While the former generates a high amount of energy through high temperatures, the latter is a non-thermal treatment that operates at lower temperatures. The temperature range for cold plasma treatment is between 25 and 65 °C [60]. The ionization of gas leads to the formation of free radicals such as ions and electrons. The composition of plasma reactive species is primarily dependent on the individual substituents of the gas that is ionized. The gases commonly used to generate plasma are argon, helium, oxygen, nitrogen and air [61].

**Figure 6:**
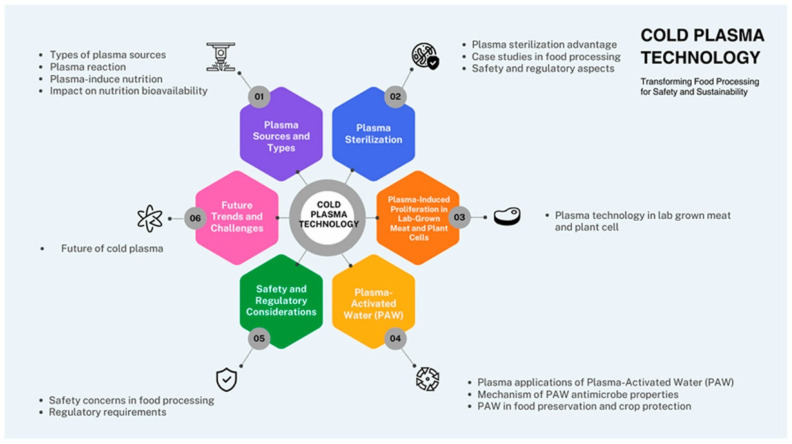
Representation of the use of cold plasma technology to transform food processing for safety and sustainability [64].

Different types of energy, including thermal, electrical, and magnetic fields, are transferred to the gases, forming the resultant plasma. Based on the constituents of this plasma, which consist of positive ions, negative ions and other reactive species [35], it has been deployed in various fields, including chemistry, chemical engineering, textiles, electronics and surface coating. Among these areas, food technology has used cold plasma to reduce the microbial load on food materials. Cold plasma has been used to enhance the physicochemical properties of food constituents, including lipids and proteins, thereby reducing the overall microbial load on or in the food [62]. This process can also sterilize food processing equipment, treat food packaging materials, and treat wastewater [63]. Since cold plasma is generated at an ambient temperature, it does not require high temperatures. Maintaining ambient temperature minimizes the risk of thermal damage to heat-sensitive food materials. Figure 6 [64] shows the applications of cold plasma technology in food processing. This technology has enabled food processing to achieve enhanced safety and sustainability.

Reactive species present in microbial cells inactivate microbes in cold plasma. This occurs through reactions between reactive species and microbes, which damage DNA, induce oxidation, and harm cellular components [65]. An example can be seen in the work of Lin et al. [66], where the use of cold nitrogen plasma prevented *Salmonella enterica* serovar Typhimurium biofilms from forming on the outer surface of an eggshell. The catabolic and anabolic activities of the bacteria were reduced by 82.2% when the samples were treated at 600 W for 2 min. Similar work has been reported by Devi et al. [67], who reduced the growth of fungal species such as *Aspergillus parasiticus* and *Aspergillus flavus* by 97.9% and 99.3%, respectively. The growth of these fungi was reduced on ground nuts when the food was treated at 60 W plasma power. Atmospheric cold plasma has also been used effectively, when combined with other gases, to reduce microbial growth. For example, Bang et al. [68] demonstrated the combination of antimicrobial washing and in-package cold plasma treatment on mandarin oranges to minimize microbial growth. It has been carried out by treating the organs at 26 kV and 27 kV for 1–4 min to inactivate *Penicillium digitatum.* The combined effect of washing the oranges in an antimicrobial solution and cold plasma reduced microbial growth without compromising other properties, such as texture, sensory, and nutritional qualities. Additionally, the treated oranges also had a reduced ripening effect compared to the untreated ones. Inactivation of *E. coli* can also be achieved using this technique, as shown by Shah et al. [69]. It also reduced the microbial load before food processing by disinfecting the surfaces of food processing equipment. Cold plasma plays a significant role in degrading pesticide residues on fresh fruits and vegetables, thereby improving food safety and maintaining product quality. The reactive species generated during the treatment process, including ions, free radicals, and electrons, interact with pesticide molecules present on the surface of the food products. These interactions lead to the breakdown of complex chemical structures into simpler, less toxic compounds through oxidative reactions. This makes it effective against a wide range of pesticides, including organophosphates and carbamates commonly found in agricultural practices. The use of cold plasma processing at mild temperatures helps preserve the nutritional value, texture, and sensory attributes of freshly produced products. This process can be directly applied to fruits and vegetables or incorporated into existing post-harvest handling systems such as washing or packaging lines. Another advantage is the reduced need for chemical decontaminants, supporting more environmentally friendly processing methods. The efficiency of pesticide degradation depends on certain factors such as treatment duration, type of gas used and power intensity. Proper optimization needs to be carried out to ensure maximum residue removal without causing undesirable changes to the developed products.

Similar to other non-thermal food processing techniques. The cold plasma method also has certain limitations. For example, one major constraint is its limited penetration depth. This makes it unsuitable for thick or dense products, as it relies on ultrasound technology. Its effectiveness can vary widely depending on food composition, surface roughness, and moisture content. The reactive species generated during plasma treatment may also lead to unintended chemical changes, such as lipid oxidation or off-flavor development. Another limitation is the lack of standardization and regulatory approval in many regions, which slows the commercialization of this process. The equipment costs and safety concerns associated with high-voltage operation also pose barriers to this process. Finally, interacting with packaging materials can alter both packaging integrity and treatment efficiency. These factors together limit cold plasma to niche applications such as the surface decontamination of fresh produce and packaging sterilization. Table 2 presents a comparative study of the performance of HPP, PEF, and cold plasma. This comparative study has been presented using consistent metrics, such as microbial log reduction, energy consumption, and nutrient impact. Each of the three food processing techniques has distinct mechanisms of action that generate these values. HPP achieves high microbial log reduction by uniformly applying hydrostatic pressure throughout the food matrix, thereby disrupting cellular membranes and protein structures in both the surface and internal regions [70]. The PEF shows a greater microbial reduction than cold plasma, due to its ability to create pores in microbial cell membranes [71]. The lower energy in the PEF process is due to the short pulse durations. The low energy requirement of the cold plasma process reflects ambient operating conditions. Nutrient degradation and surface protein modification during cold plasma processing occur through oxidative reactions involving reactive oxygen and nitrogen species [72,73].

Despite similarities in performance, these three non-thermal food processing technologies each have specific limitations for a particular food metric. For example, a key limitation of HPP is its limited effectiveness against bacterial spores and certain pressure-resistant enzymes. Some bacterial spores, such as Clostridium and Bacillus species, possess highly protective structures that allow them to survive the extreme pressure conditions used in HPP. As a result, achieving full spore inactivation often requires combining pressure with mild heat, thereby reducing the non-thermal advantage of HPP technology. In addition, many endogenous enzymes, including polyphenol oxidase and peroxidase, are relatively pressure-resistant and may remain active after treatment. This residual enzymatic activity can lead to quality deterioration during storage, including browning, off-flavor development, and nutrient loss. Consequently, HPP-treated products may still require refrigeration or additional preservation strategies.

Similar to HPP, PEF has also faced limitations due to its inefficiency in processing solid or heterogeneous foods. As the mechanism of microbial inactivation in the PEF process relies on electroporation, it requires a uniform electric field distribution throughout the food product. This condition is easily achieved in liquid or pumpable foods, but becomes problematic in solid matrices where structural complexity disrupts field uniformity. Variations in electrical conductivity between different components, such as solid particles, air pockets and liquid phases, lead to uneven energy distribution and inconsistent microbial inactivation. As a result, certain regions may remain under-processed, posing food safety risks. Additionally, solid foods cannot be easily passed through continuous PEF treatment chambers, limiting scalability and industrial application. Pretreatment steps such as size reduction or liquefaction may be required, eventually increasing processing complexity and cost.

Like HPP and PEF, cold plasma also has a major limitation: its potential to cause oxidative damage to lipids and proteins in treated foods. The reactive oxygen and nitrogen species generated during plasma treatment, which are effective for microbial inactivation, can also initiate oxidation reactions in food components. Lipid oxidation can lead to the formation of rancid off-flavors and undesirable odors, and can reduce nutritional quality, particularly in fat-rich foods. Similarly, protein oxidation may alter amino acid structures, thereby affecting functional properties and reducing digestibility. These changes can negatively impact the texture, color, and overall consumer acceptance. The extent of oxidation depends on treatment conditions such as exposure time, power level and gas composition, making process control critical. Although the optimization process can reduce these effects, complete prevention remains challenging.

## 3. Innovative Packaging Technologies

### 3.1. Microwave Technology

The use of microwave technology is essential in today’s world as a novel packaging technology. Various pieces of equipment with moisture absorption capabilities have been considered to absorb drip losses from packaged food during microwave cooking. These systems have gained widespread popularity due to their ease of operation, enabling quick, uniform food processing. It has been used in various households to reheat leftovers, but its applications in food processing are vast and varied [74]. This process operates as electromagnetic radiation with wavelengths ranging from 1 m to 1 mm. The presence of waves within the food materials vibrates the water molecules, thereby generating heat through friction. Some advantages of this technique include high speed, energy efficiency, and nutrient delivery. It has also been used for cooking and deep-fat frying, forming heterocyclic aromatic amines that can cause mutagenic changes in the body. One of the major characteristics of microwave processing (MWP) is that it can be used to process food materials on various substrates, including ceramics, metal matrix composites, fiber-reinforced plastics, alloys, and metals, as well as for material joining, coating, cladding, and material synthesis. It allows a wide range of substrates to be used for processing food under microwave technology. Starting from a strong industry, microwave heating has now predominantly penetrated the field of metal-based composites. For example, Mothibe et al. [75] reported the effects of ultrasound and microwave pretreatments on the dehydration rate and physical properties during spouted bed drying of apples. The experiments showed that ultrasound pretreatment (25.7% at 15 min) resulted in a greater loss of sugars than microwave pretreatment (1.6% at 300 W). The microwave-treated samples were harder and had lower water activity than the other pretreatment processes. Ultrasound treatment is preferred for lower-calorie fruits. Microwave heating blanching was performed at 200 W and 300 W for 1 min. A frequency of 2450 MHz and an atmospheric pressure of 1 atm were chosen as the input conditions for the experimental chamber. Four hundred grams of apple cubes was pretreated and then cooled under a high-speed fan at room temperature. Then, the samples were dried and blotted to remove excess solution and placed in a microwave-assisted spouted bed dryer. This dryer was maintained at a power and temperature of 3.5 W/g (wb) and 70 °C, respectively. The chosen conditions help decrease the rate of degradation in fruit quality. This process involved weighing the samples before and after microwave-assisted treatment.

Another work highlighting the combined use of microwave and ultrasonication processes is that by Li et al. [76]. They demonstrated the operation of the two processing methods, based on two different frequency ranges, on food materials. These combined processes have been successfully applied in food processing, including thawing, drying, frying, extraction, and sterilization. The combination of techniques, including the design of equipment and applications for processing agricultural products, has enabled reductions in nutrient degradation and energy consumption. The synergy between the heating and cavitation effects of microwave and ultrasonication processes has enhanced the overall processing efficiency of food materials by reducing damage to the product’s cell structure. The ultrasonication process has been used as an auxiliary method to enhance microwave heating efficiency, providing a pollution-free, highly efficient approach for processing food items. The explanation provided by Singh et al. [51] highlights the use of the microwave heating process from a sustainability perspective. The need for sustainability has been of utmost importance in the cutting-edge technology, industrial and competitive era. In retrospect, the microwave heating process exhibits variations in certain parameters, including efficiency, product quality, power, and time consumption, depending on the type of food material.

Although the effect of MWP has enabled researchers to drive changes in the structure, properties, and functions of macromolecular nutrients in novel foods [77], the entire heating process is very complex. The mechanisms and influencing factors of microwave technology in the development of new foods are generally beyond people’s understanding. The macromolecules in food, such as starch, lipids, and proteins, primarily determine changes in structure, characteristics, and functions under MWP. They also determine the flavor of the food materials, along with their health benefits, potential safety risks, and bidirectional allergenicity. After the introduction of microwave popcorn by the Pillsbury company in 1976, various food items, including potato chips, cakes, and noodles, have been processed using microwaves. Each of these food items possesses properties such as rapid heating, low oil content, high product quality, uniform heat distribution, and environmentally friendly production. However, it is believed that MWP will reduce the nutritional value of food and generate unknown substances with potential health risks. The gelatinization enthalpy and rheological properties are affected by microwave treatment. Temperature and enthalpy are two critical parameters for the gelatinization process. Using microwave treatment, the values of these two parameters differ across different food items. Table 3 highlights the effect of microwave treatment on starch gelatinization parameters in food. It is observed that the energy required for gelatinization varies across food species. Microwave treatment reorganizes starch molecules, resulting in tighter crystal regions. It also forces expansion and diminishes amylose dissolution, leading to a subsequent reduction in starch gelatinization. For example, microwave treatment of lotus seed starch results in a tighter crystal zone structure and reduced amylose dissolution. This causes a permanent alteration in the rheological properties of starch paste. Reducing starch gelatinization enhances the food’s overall tensile strength and formability, thereby improving the product’s crispness and strength.

The use of microwave treatment also enhances the 2,2-diphenyl-1-picrylhydrazyl (DPPH) free radical scavenging activity. This is based on the synthesis of new double bonds in products like starch, which decreases free radical reactivity. On the other hand, phenolic compounds take precedence over the starch oxidation reaction. The use of microwave heat treatment helped bind phenolic compounds in materials and increased their exposure. For example, microwave treatment increases the DPPH clearance of chestnut starch from 18.89% to 29.02% [78].

Microwave extraction technology has also emerged as an efficient technique for the recovery of essential oils and valuable bioactive compounds from plant material, particularly under dry or low-moisture conditions. By rapidly heating the material with electromagnetic waves, it disrupts plant cell structures and enhances the release of intracellular compounds. This significantly reduces extraction time compared to conventional methods, such as hydrodistillation, which are often lengthy and energy-intensive. The shorter processing time and controlled heating time help minimize the degradation of thermolabile terpenes. This preserves the aroma, flavor, and functional quality of essential oils. In addition to essential oil extraction, this approach is highly effective for recovering bioactive compounds, such as polyphenols, flavonoids, and antioxidants, from fruits, vegetables, and their processing wastes. It supports better solvent penetration and mass transfer, resulting in higher yields and improved extraction efficiency. The microwave extraction technique is particularly valuable for utilizing agricultural by-products, contributing to waste valorization and sustainable processing. Furthermore, reduced solvent usage and energy consumption make this process an environmentally friendly alternative. Optimization of power and exposure time is performed to avoid localized overheating and degradation of sensitive compounds.

There are some limitations of microwave technology that restrict its broader industrial application. A major challenge is non-uniform heating caused by uneven electromagnetic field distribution, leading to hot and cold spots within the food product. This inconsistency can compromise microbial safety and lead to quality defects in thick, dense, or irregularly shaped food products. The limited penetration depth further reduces its effectiveness for large-scale or bulk processing. The dielectric properties of food materials vary with composition, temperature and moisture, making process and predictability difficult. Additionally, some packaging materials are incompatible with microwave energy and the presence of metals can cause safety risks. Overheating in localized regions may lead to nutrient degradation, undesirable texture changes, or loss of sensory quality. The scaling up of microwave systems and process validation while maintaining uniformity remains technically complicated.

### 3.2. Ohmic Heating

This process has been a pioneering technique in food processing due to its low wall temperatures and minimal heat-transfer coefficient requirements. It also maintains the color and nutritional value of food, while providing a short processing time and higher yield [79]. It is carried out by passing an electric current through the materials to heat and process the food. With the growing popularity of heating treatment for complex foods, this process has been considered an optimized method compared to conventional thermal conduction methods. This leads to the dependence on fewer temperature gradients and avoids the limitations of inductive heating. Knowledge of the electrical conductivity of food materials and mathematical models of ohmic heating patterns is essential to carry out this technique [80]. It involves passing an alternating current through food products to generate internal heat due to electrical resistance. The electrical conductivities of different solid–liquid food materials are studied and varied in response to subsequent variations in temperature, voltage gradient, and concentration. There is a linear relationship between temperature and electrical conductivity. The basic idea is to dissipate electrical energy as heat through an electrical conductor. For example, electrical conductivity increases linearly with temperature for purely liquid foods but decreases with increasing product concentration. But with solid foods, it is more complicated, as conductivity increases linearly with temperature at low voltage gradients.

One of the major advantages of this process is rapid, uniform heating, which minimizes hot-spot-related non-uniformity and fouling. To ensure adequate safety and quality assurance protocols, it is necessary to develop mathematical models that simulate the effects of critical factors on the ohmic response. The use of ohmic heating for thawing and blanching is further advantageous because it does not generate wastewater, provides relatively uniform heating throughout the entire volume, and can be easily controlled. This process has been used to thaw shrimp blocks and frozen meat, resulting in less weight loss [81]. Ohmic heating to blanch food materials is also beneficial, as it reduces the extent of solute leaching compared to a hot water process. Researchers reduced moisture loss from blanched potato slices using electric-field-generated ohmic heating [82]. Researchers have developed certain models that can be used to calculate the heat generation rate with respect to transient energy [83]. These models have been able to detect electrical conductivity and heat generation for a static heater and a mixed fluid. In addition to the temperature and voltage gradient, the electrical conductivity of ohmic heating is also affected by porosity, hardness and bulk density.

Additionally, conductivity varies with changes in the heating of biological tissue. This is because ionic mobility changes with structural changes in the tissue. For example, the work presented by Icier et al. [80] demonstrates an overall increase in electrical conductivity with decreasing concentration. Orange juice consists of 0.2–0.6 mass fraction when heated in an ohmic manner using five different voltage gradients between 20 V/cm and 60 V/cm. The increase in conductivity ranged from 0.15 to 1.25 S/cm. To define the variation in the constituents and overall quality of the food product, research by Bozkurt et al. [84] has also tested ground beef samples with different initial fat contents. The fat contents at 2%, 9%, and 15% were cooked in an ohmic manner at 20 V/cm, 30 V/cm, and 40 V/cm, and in a conventional manner. The ohmic heating process revealed that the cooked samples were firmer than conventionally cooked samples.

Although the yield and fat retention were similar for both techniques, the reduction in volume during cooking was smaller in ohmic cooking compared to the conventional system. The voltage gradient applied during the ohmic heating process did not correlate with the quality of the cooked meat. For sterilization, Jun et al. [85] used ohmic heating to develop a reusable pouch with electrodes for long-term space missions. A 3D model was designed to ensure sterility and identify cold spots over the entire pouch. The pouch design was created to allow for reheating and sterilization of its internal contents. This process enabled optimization of electrode configurations to ensure adequate sterilization. Food thawing and blanching using the ohmic heating system has been very helpful, as this process converts approximately 90% of the electrical energy into heat. This process helps produce safe, high-quality food. A systematic study of ohmic heating in food processing is presented in Table 4 [86].

**Figure 7:**
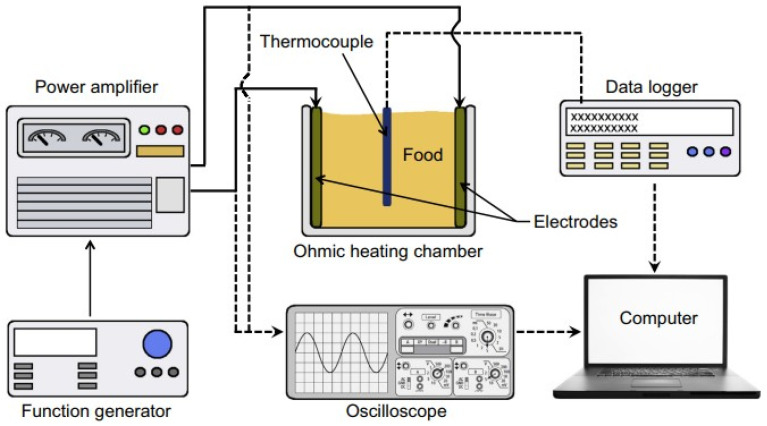
Schematic representation of the ohmic heating equipment used for food applications [92].

Figure 7 [92] represents the experimental setup used for ohmic heating of food materials. Ohmic heating has also shown significant changes in food parameters, such as microbiological inactivation, color, rheological properties, and texture. A balance is required between the microbiological safety and nutritional integrity of processed food materials [93]. Among the tested fruit and vegetable juices, ohmic heating was found to help preserve their quality. This is due to the inactivation of enzymes like polyphenol oxidase. Additionally, bacterial counts in certain vegetable juices, such as tomato juice, are reduced by ohmic heating. Among meat products, ohmic heating has also improved the quality of various products, such as chicken, meat, fish, and eggs, by reducing harmful microbes and subsequently reducing total cooking time. It has also been applied successfully in milk to reduce the microbial load. Current work is underway to implement ohmic heating at 75 °C to enhance shelf life. Different types of milk products, including flavored milk, whey, and infant formula, are being tested to assess the effects of ohmic heating on antioxidant activity and processing time. The energy required to pasteurize milk is also lower for ohmic heating than for conventional pasteurization. The ohmic heating process also has certain limitations that limit its use for liquid foods, slurries and particulate systems. Because it depends on the electrical conductivity of foods, it can only be used for foods with sufficient and uniform conductivity. Heterogeneous foods containing both solid and liquid phases may experience uneven heating due to differences in their electrical properties. Certain electrode-related issues, such as corrosion, fouling, and material degradation, may increase maintenance requirements and operational costs. Precise control of voltage and current is also necessary for safety and efficiency, especially in large-scale systems where maintaining a uniform electric field distribution is difficult. Additionally, high-voltage operation may also raise safety concerns.

### 3.3. Radio Frequency Heating

**Figure 8:**
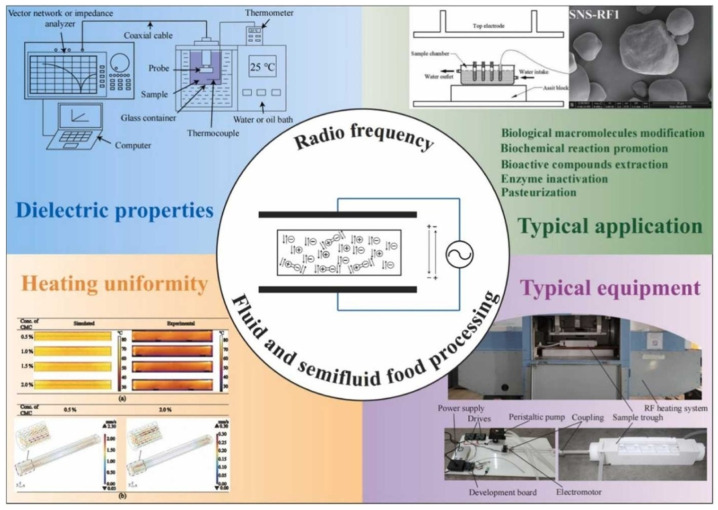
Representation of the use of RF treatment in food processing [99]. (**a**) Temperature distribution of the carboxymethylcellulose (CMC) solutions after RF heating in a horizontally placed cylindrical container (d = 60 mm and L = 600 mm) on a longitudinal surface, and (**b**) the distribution of the velocity vector and magnitude of the CMC solution after RF heating in a horizontally placed cylindrical container.

The next process has also been widely used for processing various fresh foods with high water activity and high heat sensitivity. Thermal processing of fresh foods has primarily been used to extend shelf life while avoiding chemical treatments. Radio Frequency (RF) is a promising heating technique due to its advantages, such as rapid heating, energy efficiency, superior quality retention, and environmental friendliness [94]. The heating efficiency and quality of the finished product have been optimized for various food processing applications, including microorganism reduction, disinfection, thawing, cooking, and blanching. RF is a nonionizing form of electromagnetic energy, with energy similar to that of microwave energy but with longer wavelengths [95]. This technique overcomes the bottlenecks of traditional volumetric heating methods [96]. Although this process was initially used for textile drying and glue curing in plastic and plywood processing [70], it has also been used for various food processing steps with surface heating to address non-uniform heating [97]. The RF technique has been applied at specific frequencies, including 13.56, 27.12, 40.68, 915, and 2450 MHz [98]. Figure 8 [99] showcases the use of RF treatment for processing different food materials. The RF heating process involves the generation of directional electromagnetic fields between the electrode plates. Compared to the non-uniform field in microwave ovens, RF heating produces a simpler, more uniform electromagnetic field. Two types of RF heating systems have been used in the industry: open-circuit and 50 Ω technology. While the open-circuit system generates electromagnetic power using a triode tube-integrated standard oscillator circuit, the material is heated by the electrode system of the applicator. The 50 Ω technology features an automatic tuner as part of the application circuit, which adjusts the overall impedance to match the generator’s impedance. This creates a stable coupling of RF energy and load materials during a stable heating process. Other than these two systems, the solid-state RF system has also been used in recent years. It has been able to replace traditional magnetron microwave ovens. While the traditional magnetrons operate at only one electromagnetic frequency to produce standing waves in the oven that do not migrate throughout the cavity, solid-state RF technology uses solid-state transistors to generate energy at variable frequencies. Unlike traditional microwave systems, this is a close-loop system that consists of more than one antenna to monitor the absorption rate of electromagnetic energy in the cavity and provide real-time adjustments to the RF energy output. The number of standing waves is also eliminated in the cavity to increase the preciseness of the cooked food.

The alternating electric field applied to the dielectric material induces friction between food molecules due to the movement of ions and the rotation of polarized molecules [100]. Ionic conduction predominates, with charged ions contributing more to heat generation than water molecules. This process has been utilized in various food-related applications, including defrosting [101], disinfection [102], pasteurization [103], drying [104], enzyme inactivation [105], enhancement of gelling properties [106], and modification of the internal structure of starch [107]. Different types of RF equipment for food processing applications provide uniform heating, enhance operational convenience, improve treatment efficiency, and allow customization of product quality [108]. This technique has generated heat that penetrates the samples through the rotation of polar molecules and ionic motion. This is one of the few techniques in which energy can penetrate non-metallic packaging and enable non-contact heating [109]. Continuous work is being conducted on the use of RF treatment on various food samples. For example, Hou et al. [110] used RF treatment to determine the energy consumption and product quality during RF disinfestation of coix seeds. While Tonti et al. [111] studied the RF pasteurization of milk powder, Zhu et al. [112] reported a 5.62 log reduction in *Salmonella* in liquid whole egg after RF heating. Table 5 [102] highlights the effect of moisture content on the dielectric properties of fruits and vegetables. It is observed that an increase in moisture content in an individual food product also increases the dielectric constant.

This technique has been advantageous as an alternative to conventional thermal treatment, reducing the overall undesirable effects on the food particles. The resultant food particles are not affected by the RF technique due to various factors, including changes in the development process, electrode configurations, and packaging materials used during the experimental process. For example, Shen et al. [117] highlighted the use of the RF process by identifying the optimal treatment related to the gaps between the electrode plates of the system with minimal impact on the food items. Dragon fruit slices were treated with a power, frequency, time, and temperature of 6 kW, 27.12 MHz, 10 min, and 70 °C, respectively, to determine biomechanical responses and microbial growth during a 2–3-day storage period. The food particles had a high moisture content of 82.63%, which further increased the rate of enzymatic activity and microbial growth. The total phenolics of the dragon fruits increased after RF due to the formation of new structures after processing. This is due to the easier quantification of phenolics resulting from their release from intercellular structures. The treatment of Kiwi puree under similar experimental conditions for TF delayed microbial growth and subsequently improved product quality. For these Kiwi puree products, higher concentrations of vitamin C, phenolic compounds, and antioxidant capacity were observed after RF processing. A similar study by Xu et al. [118] on buckwheat showed that the natural flora was inactivated after the RF process. When the temperature was maintained between 70 and 90 °C and the processing time was less than 20 min, the RF treatment achieved around 3 log reductions in the natural microflora. Inoculating buckwheat with pathogens and subsequently treating with a RF resulted in a further reduction of around 4 logs in pathogen levels. There was no change in color or nutrient loss after the RF treatment compared with the control samples. Apart from the advantages of RF heating, it also has certain drawbacks, including non-uniform heating due to an uneven electric field distribution. This can often lead to overheating at the edges while the interior remains under-processed. The differences in dielectric properties within food products further complicate uniform heating, particularly in heterogeneous or multi-component systems. Although RF offers deeper penetration than microwave heating, it is still insufficient for very large or dense products. Process control is challenging because heating behavior changes with moisture content, temperature, and composition. The equipment design, including electrode configuration, is complicated and costly. Additionally, the packaging materials must be compatible with RF energy, and microbial safety is inconsistent across products.

## 4. Future Research Direction

Non-thermal processing technologies have had minimal effects on the properties of food products compared to thermal treatment methods. Certain processes, such as pasteurization, evaporation, and drying, have limitations, including high energy consumption, the formation of harmful compounds as by-products, and changes in flavor and texture [119]. These processes ensure the highest standards of food safety by minimizing food processing. The experimental conditions in terms of sensory properties need to be optimized to subsequently maintain food properties, such as texture, color, taste, and nutritional value. Although the non-thermal technologies mentioned in the preceding sections have been able to inactivate microorganisms at ambient temperatures, they require specific treatment conditions, such as the use of lethal effects like high-pressure processing (HPP), pulsed electric field (PEF), ultrasound, or infrared technologies, which are related to lower pH values [15]. Additionally, the presence of certain bacterial spores and highly resistant microorganisms can render single non-thermal technologies ineffective. The effectiveness of these technologies can be further enhanced at lower process intensities by combining individual processes with conventional preservation methods. For example, Antonio-Gutiérrez et al. [120] highlighted the treatment of grape juices with ultrasonication combined with UV light to inactivate *S. cerevisiae*. There was a reduction in the microbial population in the juice by 2.8 log cycles and 0.84 log cycles after three passes of the combined non-thermal process and single UV light, respectively.

Furthermore, other parameters, such as pH, color, and °Bx, did not change after these antimicrobial treatments. Another example is highlighted by the work of Khandpur et al. [121], which demonstrates the effectiveness of combining US and UV light treatment for sterilizing juices. There was a 5 log reduction in microorganisms at 20 kHz and 100 W. Two UV lamps with individual power of 8 W and a duration of 15 min were used for the process. The inhibition of *E. coli* in apple juices, as shown by Gachovska et al. [122], was carried out using the combined use of PEF and UV methods. There was a reduction in the microorganism values to 5.35 log CFU/mL, with the PEF having an electrical field strength of 60 kV/cm and the UV treatment having a length of 50 cm and a treatment time of 2.94 s. Certain non-thermal food processing techniques, such as VFD, offer unique advantages, including the generation of intense shear forces and high mass transfer rates within a thin film, reduced processing time, and the creation of micro- and nano-sized particles for encapsulation. This cost-effective process enhances the properties of the food components while controlling particle size. These techniques also assist in creating emulsions and micro- and nano-capsules for various food applications, including the encapsulation of flavors, colors, and bioactive compounds.

The real-world implementation of the above-mentioned non-thermal technologies involves a balance between technological benefits and economic and regulatory constraints. Among the commonly used non-thermal methods, HPP is the most commercially established, mainly because regulatory authorities have already approved it. It is widely used for products such as juices, ready-to-eat meals and meat products. The popularization of HPP was accompanied by high capital expenditure (CAPEX), as industrial HPP systems require robust pressure vessels and specialized infrastructure. HPP also requires high energy consumption, thereby increasing overall operational costs (OPEX). There is also a maintenance cost and batch-mode operation, which limit throughput scalability compared to continuous systems. Despite this limitation, HPP has consistently demonstrated stronger consumer acceptance, as it is perceived as a “clean-label” preservation method that maintains food freshness. In contrast, PEF technology has strong potential for industrial-scale production due to its continuous processing capability, particularly in liquid systems. Nevertheless, the adoption of PEF remains limited to niche or pilot-scale applications due to high equipment costs and technical challenges in achieving uniform electric field distribution [123]. From a regulatory perspective, PEF-treated foods should meet stringent safety requirements, which delay the commercialization of the process [124]. The consumer perception of PEF is generally positive due to minimal processing, but awareness remains low. The inefficiency in processing solid foods further limits PEF’s industrial versatility, confining its use to liquid-processing industries. Cold plasma technology is still largely at an early stage of industrial implementation. The relatively low energy consumption and ability to operate at near-ambient temperatures make it an attractive option from an operational cost perspective. However, CAPEX can still be high due to the need for specialized plasma-generation systems and their integration into production lines [125]. Scalability also remains a major challenge, as treatment is typically limited to surfaces and achieving uniform exposure is difficult. Regulatory acceptance is still evolving due to limited standardized guidelines across regions. Consumer perception is increasingly favorable, driven by demand for chemical-free, minimally processed foods, potentially enabling premium pricing for these products.

Innovative or hybrid technologies, such as microwave, ohmic, and RF heating, are increasingly accepted and integrated into industrial food processing systems due to their compatibility with existing operations. Microwave processing is already widely used in food manufacturing for drying, cooking, and pasteurization, as it offers relatively low CAPEX and ease of integration. However, operational limitations, such as non-uniform heating and difficulty with process validation, affect scalability and product consistency. Comparatively, although ohmic heating offers much more rapid and uniform volumetric heating, it faces adaptation barriers due to electrode corrosion, safety concerns with high-voltage systems, and dependence on food electrical conductivity. These factors increase both CAPEX and OPEX, especially in large-scale systems. RF heating is primarily used in industrial drying and tempering applications. Although it offers deeper penetration than microwave and ohmic heating, the equipment’s complexity and sensitivity to product challenges limit large-scale optimization.

In terms of popularizing non-thermal technologies for futuristic food processing applications, there is an increase in the use of hybrid methods within the framework of combined technologies. As we have seen above, each of these methods has limitations: they need to be combined with mild heat, antimicrobials, or other physical treatments to enhance microbial safety and extend shelf life. The hybridization of the method also improves process efficiency. For example, combining microwave and RF heating with conventional methods helps reduce processing time and energy consumption. These synergistic approaches have become essential for overcoming the shortcomings of individual technologies. In terms of long-term effects on food matrices, each of these technologies is primarily used for its minimal impact. After processing with these technologies, subtle structural and chemical changes may occur during storage. Pressure-induced protein denaturation, electric-field-induced membrane modifications, and plasma-induced oxidation reactions can influence texture, flavor stability, and nutritional quality over time. Lipid oxidation and residual enzyme activity remain the primary concerns, especially for extended shelf-life products. It is also seen that repeated or high-intensity exposure in hybrid systems may lead to cumulative effects that are not immediately evident but manifest during storage. Standardizing processing parameters across different technologies is also needed. Variability in food composition, geometry, and physicochemical properties leads to inconsistent process outcomes, making it difficult to establish universal guidelines. Certain parameters, such as pressure level, electrical field strength, frequency, power input, and treatment time, must be optimized for each product. The lack of globally harmonized regulatory standards and validated process models slows industrial adoption and complicates safety assurance.

From an academic perspective, the future of the non-thermal food processing technologies will depend on their ability to integrate into multi-hurdle systems. Advancements in sensor technologies, process modeling, and automation should enable better real-time control and greater standardization. Sustainability pressure will always favor energy-efficient, waste-reducing technologies, thereby giving non-thermal approaches a strategic advantage. A better adoption will not only require technical refinement of individual processing technology, but also clearer regulatory frameworks and improved consumer communication. In the long term, these processing technologies are likely to become mainstream solutions for producing environmentally sustainable, high-quality food products.

## 5. Conclusions

Although some of these inactivation methods have been effective and demonstrated the capabilities of non-thermal processes, the understanding of the combined effects of these technologies and their feasibility under various ambient conditions remains unclear. An increase in the antimicrobial effect on a specific microbial cell can be achieved by simultaneously optimizing both the treatment conditions and the self-repair mechanism. This will increase external energy, placing the microbial cells in a sublethal state. Cellular metabolism can be repaired after the disappearance of a hostile environment. The combination of non-thermal technologies for sterilization can also target key components, such as membrane proteins, lipids, and DNA. This will not only disturb several functions of the microbial cell but also inhibit the self-repairing process [126]. Compared with single treatments in food processing, combined non-thermal methods offer additional advantages beyond inactivation, including improved product quality, extended shelf life, reduced dependence on chemical preservatives, and retention of nutrients.

## Figures and Tables

**Table 1:** Characteristics of thermal and non-thermal food processing technologies.

Parameter	Thermal Processing	Non-Thermal Processing
Primary mechanism	Application of heat	Other forms of energy like pressure, electricity and radiation
Heat sensitivity	Food is subject to heat	Heat-sensitive components are preserved
Microbial inactivation	Destroys microorganisms	Inactivates microorganisms and enzymes
Enzyme inactivation	Inactivates enzymes	Preserves enzymes or inactivates them
Sensory changes	Possibility of affecting sensory characteristics	Minimizes sensory changes and preserves nutritional value
Shelf life	Extended shelf life	Extended shelf life
Impact on nutrients	Possibility of degradation of nutrients	Minimized degradation of nutrients
Food quality	Possibility of change in texture, flavor, and color	Preservation of nutritional quality
Application	Suitable for a wide range of applications	Suitable for heat-sensitive food and those seeking fresh-like qualities

**Table 2:** Comparison between the performances of HPP, PEF and cold plasma using consistent food processing metrics.

Parameter	High-Pressure Processing (HPP)	Pulsed Electric Field (PEF)	Cold Plasma
Microbial log reduction	Up to 5 log reduction	Typically, 3–5 log reduction in liquids, with possible regrowth during storage	Typically, 2–4 log reduction, mainly surface decontamination
Energy consumption (kWh/kg)	~1.5–3 kWh/kg	Lower than HPP, energy savings up to 18%	~0.5–2 kWh/kg (energy-efficient but variable)
Vitamin retention	High retention (>90% vitamin C), excellent stability during storage	High retention, sometimes slightly better immediate retention than HPP	Generally good retention due to non-thermal nature, but oxidation risks exist
Protein denaturation	Moderate, pressure-induced structural changes without severe degradation	Minimal denaturation, mild heating may occur	Possible surface protein oxidation/denaturation due to reactive species
Penetration depth	Uniform throughout the product (bulk treatment)	Effective in liquids (uniform field in flow systems)	Limited (surface treatment only)
Process type	Batch	Continuous	Batch/inline surface treatment
Industrial maturity	Highly commercialized	Emerging, limited scale-up	Emerging, limited standardization

**Table 3:** Effect of microwave treatment on starch gelatinization parameters in food.

Food Species	Gelatinization Temperature (°C)	ΔH (J/g)	Ref.
Maize	10.69	3.1	[9]
Chinese chestnut	2.79	2.49	[9]
Barley	12.7	2.8	[36]
Indian horse chestnut	20.18	1.4	[14]
Peanut	11	2.3	[37]
Millet	7.83	5.78	[25]

**Table 4:** Comparison between the study on the use of ohmic heating in processing different food products [86].

Application	Electrical Properties	Temperature (°C)	Time (min)	Overall Outcomes	Ref.
Black garlic pretreatment	110, 120 and 130 V (Voltage); 5.38 S/m (EC); 11, 12, 13 V/cm (Electric Field Strength/EFS)	60,70, 80	10, 15, 20	A combination of 130 V at 70 °C for 10 min resulted in a higher reduction in sugar concentration (3.7 ± 0.02 mg/g) in the samples. It reduced the time required to attain the characteristic black garlic color from 30 to 12 days.	[87]
Carrot juice pasteurization	60 kHz (AC); max, 120 V/cm	15–80	7	The ohmic-heated juice was preferred for color (*p* < 0.05) and was well accepted by consumers.	[88]
Inactivation kinetics (Weibull model) and morphological changes in *Salmonella* spp. In infant formula	60 kHz; 6 V/cm	50, 55, 60	45, 24, 5	Combination of 130 V at 60 °C, OH showed a higher inactivation rate than conventional heating, improving nutritional value and food safety.	[89]
Liquid egg pasteurization (combined with concurrent external heating)	20 kHz; 50 V	20–63.3	1.5–3.5	Ohmic heating treatment, combined with concurrent external heating, reduced process time and prevented local overheating.	[90]
Pre-cooking meatballs	15.26 V/cm; 1.5–2.25 S/m	20–75	1.5	Ohmic heating increased cooking yields, reduced the total number of mesophilic aerobic bacteria, mold, yeast, and Staphylococcus aureus, and eliminated these species.	[91]

**Table 5:** Dielectric properties of fruits and vegetables at selected temperatures in the RF range [102].

Product	Temperature (°C)	Moisture Content (%)	Dielectric Constant		Loss Factor		Penetration Depth (m)		Ref.
			27.12 MHz	40.68 MHz	27.12 MHz	40.60 MHz	27.12 MHz	40.60 MHz	
Red delicious apple	20	87	70.6	70.8	130.7	87.5	0.189	-	[113]
	40		66.8	66.8	178.6	119.9	-	-	
	60		81.9	79.9	135.5	88.7	-	-	
Apricots (dried)	20	24.6	33.9	32.3	11.8	10.6	0.888	0.649	[114]
	40		37.4	35.7	19.9	16.2	0.563	0.45	
	60		40.8	38.9	37.4	28.9	0.328	0.274	
Dates	20	19.7	27.2	25.5	10.1	9.0	0.93	0.682	[114]
	40		31	28.9	15.0	12.2	0.675	0.539	
	60		35	32.9	26.9	20.0	0.414	0.357	
Figs (dried)	20	27.3	37.7	35.7	14.4	13.1	0.767	0.555	[114]
	40		42.3	40.1	23.8	19.2	0.50	0.405	
	60		46.5	44.2	42.2	32.7	0.31	0.257	
Grapefruit (pulp)	20	-	99.42	-	245.7	-	-	-	[115]
Kiwi slices (air-dried)	20	60.5 70.2	81.49	74.12	332.14	235.17	0.771	0.642	[116]
	80		90.68	82.98	777.16	546.03	0.473	0.389	
	20		97.53	88.62	407.45	284.32	0.694	0.583	
	80		96.74	86.06	808.19	576.96	0.464	0.378	

## Data Availability

No new data were created or analyzed in this study.
